# Grasshopper Community Response to Climatic Change: Variation Along an Elevational Gradient

**DOI:** 10.1371/journal.pone.0012977

**Published:** 2010-09-23

**Authors:** César R. Nufio, Chris R. McGuire, M. Deane Bowers, Robert P. Guralnick

**Affiliations:** 1 Department of Ecology and Evolutionary Biology, University of Colorado, Boulder, Colorado, United States of America; 2 University of Colorado Natural History Museum, University of Colorado, Boulder, Colorado, United States of America; 3 The Environmental Studies Program, University of Colorado, Boulder, Colorado, United States of America; Umea University, Sweden

## Abstract

**Background:**

The impacts of climate change on phenological responses of species and communities are well-documented; however, many such studies are correlational and so less effective at assessing the causal links between changes in climate and changes in phenology. Using grasshopper communities found along an elevational gradient, we present an ideal system along the Front Range of Colorado USA that provides a mechanistic link between climate and phenology.

**Methodology/Principal Findings:**

This study utilizes past (1959–1960) and present (2006–2008) surveys of grasshopper communities and daily temperature records to quantify the relationship between amount and timing of warming across years and elevations, and grasshopper timing to adulthood. Grasshopper communities were surveyed at four sites, Chautauqua Mesa (1752 m), A1 (2195 m), B1 (2591 m), and C1 (3048 m), located in prairie, lower montane, upper montane, and subalpine life zones, respectively. Changes to earlier first appearance of adults depended on the degree to which a site warmed. The lowest site showed little warming and little phenological advancement. The next highest site (A1) warmed a small, but significant, amount and grasshopper species there showed inconsistent phenological advancements. The two highest sites warmed the most, and at these sites grasshoppers showed significant phenological advancements. At these sites, late-developing species showed the greatest advancements, a pattern that correlated with an increase in rate of late-season warming. The number of growing degree days (GDDs) associated with the time to adulthood for a species was unchanged across the past and present surveys, suggesting that phenological advancement depended on when a set number of GDDs is reached during a season.

**Conclusions:**

Our analyses provide clear evidence that variation in amount and timing of warming over the growing season explains the vast majority of phenological variation in this system. Our results move past simple correlation and provide a stronger process-oriented and predictive framework for understanding community level phenological responses to climate change.

## Introduction

Over the last several decades, global surface temperatures have increased and this warming pattern has emerged as one of the most pressing environmental issues affecting global ecosystems. One major effect has been the alteration of the phenology of a variety of plants and animals [1], [2], [3]. Numerous phenological studies have shown, for example, an earlier start of the growing season across the northern hemisphere [4] and that spring- and summer-associated events, such as first flowering periods and first appearances of insects, mammals and plankton blooms are occurring earlier than in previous decades [2], [5], [6]. This phenological advancement has also affected numerous bird species that are migrating earlier than previously recorded [7]. While warming has affected the phenology of many taxa, community-level studies have found that not all species display a phenological advancement, with nearly 25% remaining stable or, less often, displaying phenological delays [8], [9] (see also [1]).

Community-level approaches to understanding the impacts of climatic change allow for a better understanding of the degree to which different members of a community are being affected [1], [9] and to determine which species groups are most susceptible to warming temperatures [10], [11]. Studies on lake plankton, for example, showed that fast-growing, early species are better able to track warming patterns than later-appearing species that display slow-growing and more complex life-history strategies [12]. Likewise, dragonfly species on the wing in spring tended to exhibit a greater advance in phenology than those appearing later in the summer [13]. That early emerging species are more likely to track warming patterns than later species has also been found for plants [14], [15].

Whether species within communities display phenological advancement may depend on the degree to which the temperature experienced by a community has changed [16]. For example, those species living at higher northern latitudes, where warming has been documented to be the most dramatic, have shown the greatest increase in phenological advancement and the most extensive range shifts [3], [17]. Similarly, communities occurring along an elevational gradient may show differences in levels of advancement that also reflect variation in the amount of warming that they experience [18], [19].

In this study, we present an ideal system that utilizes past (1959–1960) and present (2006–2008) surveys of four grasshopper communities found along an elevational gradient in the Front Range of northern Colorado to explore the effects of climate changes on grasshopper phenology.

More specifically, we use daily temperature records from the past and present at all sites to quantify how the amount and timing of warming associated with each site, and changes in growing degree day (GDD) accumulation rates (a measure of heat input relevant to organisms) affect the timing to adulthood of grasshoppers. Grasshoppers are an excellent model group with which to study the impacts of climate change on phenology because of their sensitivity to thermal environments [20]. Thermal conditions determine the distribution patterns of grasshoppers and other insects and affect such traits as developmental time and rates of water loss, adult size, digestive efficiency and even ability to avoid predators [21], [22]. In addition, variation in temperature affects the life-history traits of grasshoppers, such as clutch sizes and egg mass [23], [24], and extreme temperatures may be associated with demographic changes that could lead to grasshopper outbreaks [25], [26].

This resurvey of four prairie to subalpine communities that differ in their amount of warming (McGuire *et al*., *in review*), but not in day length, allowed us to directly test the hypothesis that warming is affecting the phenology of grasshopper communities. As well, availability of past and present daily temperature data at each site allowed us to first establish whether the required GDDs to reach adulthood are similar across the two surveys and then to determine whether phenological advancements (or lack thereof) may be explained by changes (or a lack of changes) in seasonal GDD accumulation patterns. We also examined whether species that reach adulthood early versus late in the season differ in their degree of phenological advancement and whether this difference might be explained by the specific timing of warming. Finally, as both season length shortens and average seasonal temperatures decline with increases in elevation, we also investigated how the number of GDDs required to reach adulthood changes with elevation.

## Materials and Methods

### Study sites and weather data

We resurveyed the grasshopper communities at four sites in the Front Range of northern Colorado that were originally sampled 50 years ago on a weekly basis during the field seasons of 1959 and 1960 [27]. The four resurveyed sites are referred to as Chautauqua Mesa (1752 m; 39°99′N–105°285′W), A1 (2195 m; 40°015′–105°376′), 3.9 km west of B1 (hereafter referred to as B1; 2591 m; 40°0219′–105°453′), and C1 (3048 m; 40°036′–105°547′). The habitats at these sites are all grassy clearings associated with prairie, lower montane, upper montane, and subalpine forests, respectively.

Three of the four Alexander and Hilliard survey sites were associated with long-term weather stations referred to as A1, B1 and C1. These weather stations were established in 1952 and designed to collect daily temperature data [28], which they continue to do. These weather stations are currently serviced by the Niwot Ridge Long-Term Ecological Research Project and the University of Colorado Mountain Research Station. The fourth site, Chautauqua Mesa, was established as a protected area in 1898 and is currently managed by Boulder City Open Space. For long-term climate data associated with Chautauqua Mesa we used the United States Department of Commerce's National Oceanic and Atmospheric Administration (NOAA) weather station (Cooperative ID 050848) currently in Boulder, Colorado (1672 m; 39°59′31–105°16′00). This weather station is located 1.3 km away from Chautauqua Mesa and at a similar elevation (Chautauqua Mesa, 1752 m).

At all sites, we quantified and statistically compared, using a t-test, yearly mean seasonal temperatures (March 1 to August 31) over the last decade (1999–2008) to yearly mean seasonal temperatures during the decade containing Alexander and Hilliard's original study (1955–1964). March was chosen as start of the season because it is typically the month where temperatures first exceed 12°C at the lower sites (see Growing Degree Day calculations below) and August 31 was chosen because a majority of individuals of all focal species at each site have become adults by then (detailed climate methodology and data available at http://ghopclimate.colorado.edu/). The goal of this analysis was not to summarize the complexities of how climate has changed at these sites during the last 50 years (we address this in a separate study, McGuire et al., *in review*), rather it was to compare the temperatures that existed during the field seasons when Alexander collected and during the recent study. This comparison was used to determine which sites showed the greatest changes in seasonal temperatures and thus which sites are most likely to be associated with changes in grasshopper phenology. Because some years at some sites were associated with missing months or years of weather data, the following years at certain sites were omitted from the comparison of the decades around Alexander's and our current resurvey; 1959 and 2003 from A1 and 1956, 2001, 2002, 2003 from B1.

### Previous and current grasshopper surveys

The Alexander Collection, which is housed at the University of Colorado's (CU) Natural History Museum, is composed of approximately 24,000 pinned and labeled grasshoppers collected during the 1930's to the 1960's from the Rocky Mountain and plains regions of Colorado. During 1958 to 1960, Gordon Alexander processed over 65,000 grasshoppers as he and his team repeatedly sampled numerous sites along the Front Range of Colorado. During the 1959–1960 portion of the survey, Alexander surveyed several field sites (including Chautauqua Mesa, A1, B1 and C1) on a weekly basis to examine the phenology of grasshoppers along a prairie to sub-alpine elevational gradient near the fortieth parallel [27]. In addition to the 11,000 specimens that make up part of this voucher collection, the complete 1958–1960 survey data are available in detailed field notebooks that include information on the life stage, sex, species abundances and diversity of all grasshoppers collected during each sampling event. The survey data in these notebooks were used to reconstruct the timing to adulthood of grasshoppers during the 1959–1960 survey.

The current grasshopper survey resampled the grasshopper communities associated with four of Alexander and Hilliard's [27] main collecting sites, Chautauqua Mesa, A1, B1 and C1.

Grasshopper communities associated with sites B1 and C1 were resurveyed during the 2006 to 2008 field seasons and those associated with Chautauqua Mesa and A1 were resurveyed in 2007 and 2008. Beginning in mid-May at Chautauqua Mesa and A1, late-May at B1 and early June at C1, we conducted weekly surveys of the grasshoppers at each site. We used the same collecting protocol as used in Alexander and Hilliard's [27] original study, which consisted of 1.5 person-hour of sweep netting (divided among 1 to 3 surveyors) and 0.75 person-hours of searching for adults and juveniles that may have been missed by sweep netting. All collected specimens were identified to species and their developmental stages recorded. In addition, voucher specimens from each collecting event were pinned, labeled and added to the CU Museum of Natural History collection. To ensure that the earliest sampled adults of each species were residents of a particular site, as opposed to “accidentals” that might have been blown in from lower elevations [29], the first occurrence of all adults at a site was verified by determining whether late instar individuals were also present. Using this method to screen for accidentals, only one first adult occurrence date was modified because of the collection of an accidental; the adult emergence of *Circotettix rabula* was changed from July 2 to July 15 in 2007 at B1.

The grasshopper species used in this study were those that were present during both the 1959–1960 and current surveys (Table 1). In addition, we used only those species that diapause over the winter as eggs because Alexander's original survey missed the early timing to adulthood of nymphal diapausers at all sites. Nymphal diapausers are grasshoppers that become adults in early spring, lay eggs that hatch in late summer and have juveniles that overwinter as 3rd to 5th instars. Egg diapausers typically become adults in mid to late summer and lay eggs that will not hatch until the following year. All species in this study are also univoltine (Alexander and Hilliard 1969, pers. obs.). For a complete list of the species associated with each of the surveyed sites and their life-histories, see Alexander and Hilliard [27].

**Table 1:** The average number of individuals collected of each species at each site during the 1959–1960 surveys and the relative increase or decrease in abundance during the recent surveys.

Stations Species	Average 1959–1960*	Difference relative to 1959–1960		
		2006	2007	2008
Chautauqua Mesa				
* Aeropedullus clavatus*	233	—	−123	−148
* Melanoplus confuses*	36	—	−23	−20
* Melanoplus sanguinipes*	139	—	−100	−108
* Melanoplus bivittatus*	189	—	94	453
* Melanoplus dawsoni*	57	—26	−6	
* Hesperotettix viridis*	83	—	−42	−26
1959–1960 Seasonal average:	737			
Station A1				
* Aeropedullus clavatus*	10	—	12	19
* Melanoplus confuses*	67	—	−52	23
* Melanoplus dodgei*	146	—	−79	−56
* Melanoplus sanguinipes*	256	—	−217	−175
* Cratypedes neglectus*	165	—	−122	−54
* Camnula pellucida*	64	—	−46	−40
* Hesperotettix viridis*	127	—	−88	−53
* Melanoplus bivittatus*	37	—	−23	−4
* Trimerotropis cincta*	66	—	−58	−50
1959 Seasonal total:	938			
3.9 km West of Station B1				
* Aeropedullus clavatus*	206	−91	−120	−59
* Melanoplus dodgei*	244	−163	−149	−102
* Camnula pellucida*	91	−62	−74	−37
* Circotettix rabula*	10	11	0	37
* Melanoplus dawsoni*	111	−32	−78	−40
* Melanoplus packardii*	12	332	104	259
* Chloealtis abdominalis*	10	2	−1	13
1959–1960 Seasonal average:	682			
Station C1				
* Melanoplus dodgei*	90	95	−7	22
* Melanoplus fasciatus*	83	−14	24	−41
* Camnula pellucida*	83	893	−16	48
* Chloealtis abdominalis*	15	64	0	24
1959–1960 Seasonal average:	271			

*for A1, species abundance data only reflects the 1959 collection.

We determined whether day of year of first adult appearances has changed over the last fifty years using a paired t-test approach. In particular, at each site we compared the earliest day of adult appearance of each focal species in the 1959–1960 survey with the average date to adulthood during the 2006–2008 survey. The first appearance of each species during the 1959–1960 survey was used as this would lead to a more conservative estimate of advancement in the time to reach adulthood than had we used a mean value. That is, in order to be considered a significant advancement, grasshoppers in the new resurvey would have to exceed the earliest timing to adulthood associated with the 1959 to 1960 surveys. Because survey data in 1960 for A1 contained several important sampling gaps that may have missed the first adults for several species, we only compared the time to reach adulthood in 1959 to the 2007–2008 survey data for this site. As Alexander and Hilliard collected on a weekly basis at each site, advancement in phenology for a species in the current survey must exceed Alexander and Hilliard's seven day sampling window to be considered at least marginally earlier (or later) than that found in the original study. We did not explore changes in hatching times in this study because we found that many of the original 1959–1960 surveys lacked the sample sizes for the earliest instars that would be required to determine when first hatching might begin.

We also explored whether species that became adults early or late in the season during Alexander and Hilliard's original survey experienced larger phenological advancements in the new survey. For this analysis, we used a general linear model to regress total phenological advancement (the difference in the number of days required to reach adulthood for each species between that of the original survey and the recent surveys) with the earliest day of year to reach adulthood during the original survey as a continuous variable and survey year (2006, 2007, 2008) as a categorical variable. No significant interaction effects were found in these analyses and so are not reported. Finally, we used Spearman rank correlations to determine whether a species' advancement, lack of advancement or delay in advancement could be explained by a relative decrease or increase in a species' abundance over the last 50 years. In these analyses, we regressed, across all sites and years and within all sites and years, changes in the relative abundance of species in the current surveys (2006–2008) relative to their 1959 to 1960 averages (Table 1) with our recorded measures of phenological advancement (Table 2). As our current sampling dates have exceeded those used during the 1959–160 surveys, seasonal totals were calculated using only the sampling periods that overlapped between both studies. Thus, seasonal totals include the abundance of species from May 15 to September 20 for Chautauaqua Mesa, from May 15 to September 20 for A1, from May 15 to September 26 for B1 and from June 1 to September 7 for C1. Nonparametric statistics were used in these analyses because changes in relative abundance were not normally distributed and could vary by up to two orders of magnitudes.

**Table 2:** Grasshopper communities, phenological advancements and GDDs.

Station Species	Earliest day of year of 2006	Change in timing to adulthood			GDDs*
adult appearance (1959–1960)		2007	2008	(±SE)	
Chautauqua Mesa (1752 m)					
*Aeropedullus clavatus*	152	—	**−11**	*4*	232+
*Melanoplus confusus*	155	—	*0*	*1*	274+
*Melanoplus sanguinipes*	176	—	*7*	*2*	507 (27)
*Melanoplus bivittatus*	181	—	*2*	**−9**	548 (14)
*Melanoplus dawsoni*	186	—	*−5*	*−1*	580 (18)
*Hesperotettix viridis*	186	—	*−3*	*−4*	580 (18)
Station A1 (2195 m)					
*Aeropedullus clavatus*	167	—	**−10**	*−3*	190
*Melanoplus confusus*	167	—	**8**	*−3*	190
*Melanoplus dodgei*	174	—	**−17**	**−16**	240
*Melanoplus sanguinipes*	183	—	*7*	*1*	292
*Cratypedes neglectus*	195	—	**−26**	**−19**	386
*Camnula pellucida*	195	—	**10**	*3*	386
*Hesperotettix viridis*	202	—	**−12**	*−4*	433
*Melanoplus bivittatus*	202	—	*3*	**−11**	433
*Trimerotropis cincta*	202	—	*3*	*−4*	433
3.9 km West of Station B1 (2591 m)					
*Aeropedullus clavatus*	172	**−13**	**−17**	**−10**	137 (19)
*Melanoplus dodgei*	172	**−13**	**−17**	**−10**	137 (19)
*Camnula pellucida*	202	**−14**	**−** *5*	**−11**	289 (6)
*Circotettix rabula*	207	**−19**	**−10**	**−16**	315 (20)
*Melanoplus dawsoni*	215	**−27**	**−18**	**−8**	367 (6)
*Melanoplus packardii*	216	**−28**	**−26**	**−18**	389 (16)
*Chloealtis abdominalis*	216	**−21**	**−19**	**−18**	389 (16)
Station C1 (3048 m)					
*Melanoplus dodgei*	182	**−10**	*−5*	*−3*	61(1)
*Melanoplus fasciatus*	202	**−15**	*−3*	*−5*	111 (15)
*Camnula pellucida*	209	**−22**	**−10**	***−*** *3*	144 (26)
*Chloealtis abdominalis*	216	**−16**	**−17**	**−10**	159 (26)

*for A1, only GDD values for 1959 are provided.

+ Only GDDs for 1960 available and provided.

Time to first appearance of adults during 2006 to 2008 at the four resurvey sites compared to the first day of adult appearance in 1959–1960. Species are arranged within sites from those that reach adulthood earlier to later in the season. Negative numbers reflect advancements in the days to adulthood, while positive numbers reflect a relative delay in timing to adulthood. Bolded numbers reflect periods that exceed at least a sampling week between the previous and current surveys. The number of growing degree days (GDDs) required by the species during the 1959 to 1960 survey are also shown.

### Growing degree day accumulation

Growing Degree Days (GDDs) are a measure of the physiological time that is required for ectotherms to complete a given developmental stage [30]. The GDDs required to reach a given developmental stage are measured as accumulated daily heat units above a specified base temperature (below which development does not occur) and below a thermal maximum (above which development ceases) [31]. In this study, GDDs were calculated using the single-sine growing degree day method with a fixed spacing of 12 hours between daily maximum and minimum temperatures [32]. A single-sine wave function was chosen as the basis of our degree day calculations because, given only daily maxima and minima data, a sine function tends to reflect the actual temperatures throughout a day more accurately than a simple triangle function[33].

The lower temperature limit for grasshoppers has been shown to be between 10 and 17 degrees C [34], [35], [36]. We used a lower temperature threshold of 12°C for degree day calculations because in a preliminary analysis where we used 10° to 17° as threshold minima, 12°C produced GDDs values (associated with when each species became adults) that were most similar across the 1959 and 1960 surveys at Chautauqua Mesa, B1 and C1 (Nufio, *unpublished data*). The GDD differences between the 1959 and 1960 values at each site were determined by calculating the GDDs associated with when each grasshopper species reached adulthood given different threshold minima, subtracting the highest predicted value from the lowest value and totaling up this difference across the species within a site. A high temperature cut-off threshold was set at 38°C, a value thought to be associated with heat stress in grasshoppers [21], [35]. However, as temperatures at all four sites did not reach 38°C during the previous and recent surveys, this upper temperature threshold did not play a role in calculating GDD values.

To determine whether the advancement of a species' phenology (when advancement occurred) was due to changes in the rate at which degree days were accumulated at each site, we regressed the average GDDs associated with the timing to adulthood for each species at each site during the 2006–2008 survey against the average GDDs required for each species within the site to reach adulthood during Alexander and Hilliard's original 1959 to 1960 survey (except for A1 where, due to the lack of survey data in 1960, only the 1959 survey data were used). A slope not significantly different from one would suggest that the required GDDs are similar between both surveys. If GDDs associated with the timing to adulthood remained similar during both surveys but the timing to adulthood changed, phenological change could then be attributed to changes in GDD accumulation patterns between years. We also used the average required GDDs to adulthood at each site and a linear regression to explore the degree to which the GDDs required by communities decrease along this elevational gradient.

Finally, to determine how GDD accumulation patterns varied in our current survey years relative to Alexander and Hilliard's original survey, for all sites we first averaged the accumulated GDDs associated with each day, from March 1st to August 31st, using the 1959 and 1960 climate data. We then subtracted the mean number of GDDs associated with each day of year during the 1959–1960 survey from the number of GDDs accumulated during that same date during the 2006 to 2008 survey years. This running difference allowed us to determine the degree to which the GDD accumulation patterns differed across the studies as well as when the differences become most pronounced in time.

## Results

### Changes in seasonal mean temperature

**Figure 1:**
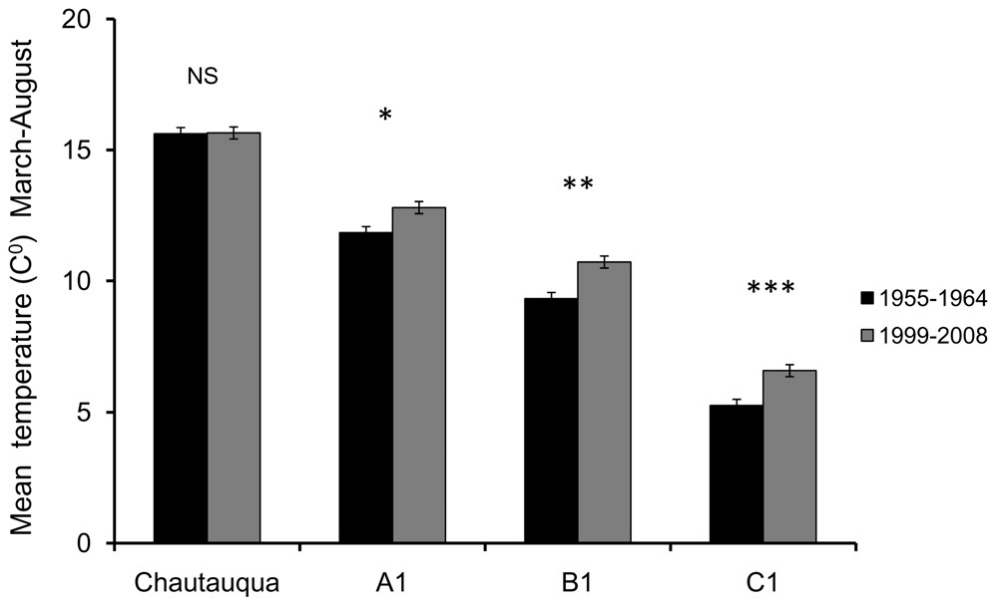
Seasonal warming at the four survey sites. Ten-year mean temperatures from March through August across the survey sites for the years during Alexander's original survey (1955–1964) and the years including and prior to the resurvey (1999–2008). NS =  Not Significant, * P = 0.01, ** P = 0.001, *** P = 0.0002.

An analysis of the daily mean temperatures experienced at the surveyed sites during 1999 to 2008, relative to those experienced by the sites during 1955–1964, showed that changes in warming across these two time periods were elevation dependent. No difference was found in the mean yearly March through August temperatures between the original and recent survey periods at Chautauqua Mesa (t_1, 19_  = 0.12, P = 0.90; Figure 1). However, mean March through August temperatures were found, on average, to be 0.96, 1.40 and 1.33C° warmer at A1 (t_1, 17_  = −3.04, P<0.008), B1 (t_1, 15_  = −3.96, P<0.001) and C1 (t_1, 19_  = −4.75, P<0.0001), respectively (Figure 1).

### Time to reach adulthood

Fourteen species of grasshoppers were found in sufficient abundance during both sampling periods to be included in this analysis (Table 2). Changes in the time to reach adulthood were site dependent (Table 2). At the lowest site, Chautauqua Mesa, there was not a consistent, discernable pattern of change in grasshopper time to adulthood but a paired t-test detected a marginal shift towards an earlier advancement (t_1,5_ = −2.55, P = 0.05; Table 1). At this site, however, time to adulthood of only two of the six focal species, *Aeropedellus clavatus* in 2007 and *Melanoplus sanguinipes* in 2008, exceeded the 7 day sampling window that was established by Alexander and Hilliard's (1969) original survey. Thus, the marginal differences between the 1959–1960 survey and the present-day survey appears to be the result of current samples being collected earlier within a sampling week than previously, and therefore likely represent vagaries of sampling as opposed to a real difference in advancement to adulthood.

Changes in the time required to reach adulthood at Station A1 were not consistent or easy to categorize and a paired t-test did not detect an overall phenological advancement (t_1,8_ = −5.24, P = 0.14). Of the nine focal species in the 2007 survey, three species did not show changes in the time to reach adulthood (they were within the 7 day sampling window), two species showed a marginal advancement (10 to 12 days earlier than in the original study), two species showed a notable advancement (17 to 26 days earlier than in the original study) and two species became adults slightly later than expected (by 8 and 10 days) (Table 2). While *M. dodgei* and *Cratypledes neglectus* continued to show an advancement to adulthood in 2008, no other species showed a significant advancement in both years. In 2008, *M. bivittatus* also showed an advancement of 11 days while the six other species remained stable.

At B1, all sampled species showed a striking advancement in the time to reach adulthood and this was detected when the earliest timing to adulthood during the 1959 to 1960 surveys and the average of the 2006 to 2009 surveys were compared (t_1,6_  = −7.76, P = 0.0002). In 2006, grasshoppers at this site became adults, on average, 19 (±2.4 SE) days earlier than they had 50 years prior (Table 2). In 2007 and 2008, grasshoppers became adults earlier than previously by an average of 16 (±2.5) and 13 (±1.6) days, respectively. Controlling for the effects of survey year (2006, 2007, 2008), the changes in timing to adulthood by a particular species was significantly affected by the time of the season (day of year) when that species became an adult during the original survey (F_1,18_ = 5.41, P = 0.03). That is, species that typically become adults later in the season experienced a greater advancement than species that become adults early in the season. In this analysis, a significant year effect was detected (F_2,18_ = 3.51, P = 0.04), indicating that the overall level of phenological advancement differed significantly across years.

While grasshopper species at C1 did show an advancement in their timing to adulthood (a pattern detected when comparing the previous and current surveys; t_1,3_ = −5.24, P = 0.01), this was not as dramatic as at B1. At C1, in 2006, 2007, and 2008, the grasshopper communities advanced their timing to adulthood by 15.75 (±2.5), 8.75 (±3.1), and 5.25 (±1.7) days, respectively. It appeared that, over time, the advancement of species across years declined, such that all species showed an advancement in 2006 while only one species showed an advancement in 2008 (Table 2). As at B1, when controlling for the effects of year (2006, 2007, 2008), changes in advancement to adulthood by a particular species were explained by the time of the season (day of year) that that species originally became an adult during the original survey (F_1,15_ = 7.95, P = 0.02). In this general linear model, as at B1, year was significant (F_1,15_ = 16.22, P = 0.003).

Finally, the hypothesis that differences in phenological advancements could be explained by relative changes in the abundance of species over the last 50 years was not supported. The relationship between changes in species abundance and phenological advancement was not detected when pooling data across all sites and years (Spearman ρ = −0.14, P = 0.26), nor when examining these relationships within the surveyed sites (Chautauqua Mesa, Spearman ρ = −0.30, P = 0.34; A1, Spearman ρ = 0.06, P = 0.82; B1, Spearman ρ = −0.35, P = 0.12; C1, Spearman ρ = −0.21, P = 0.51).

### Growing degree day accumulation patterns

**Figure 2:**
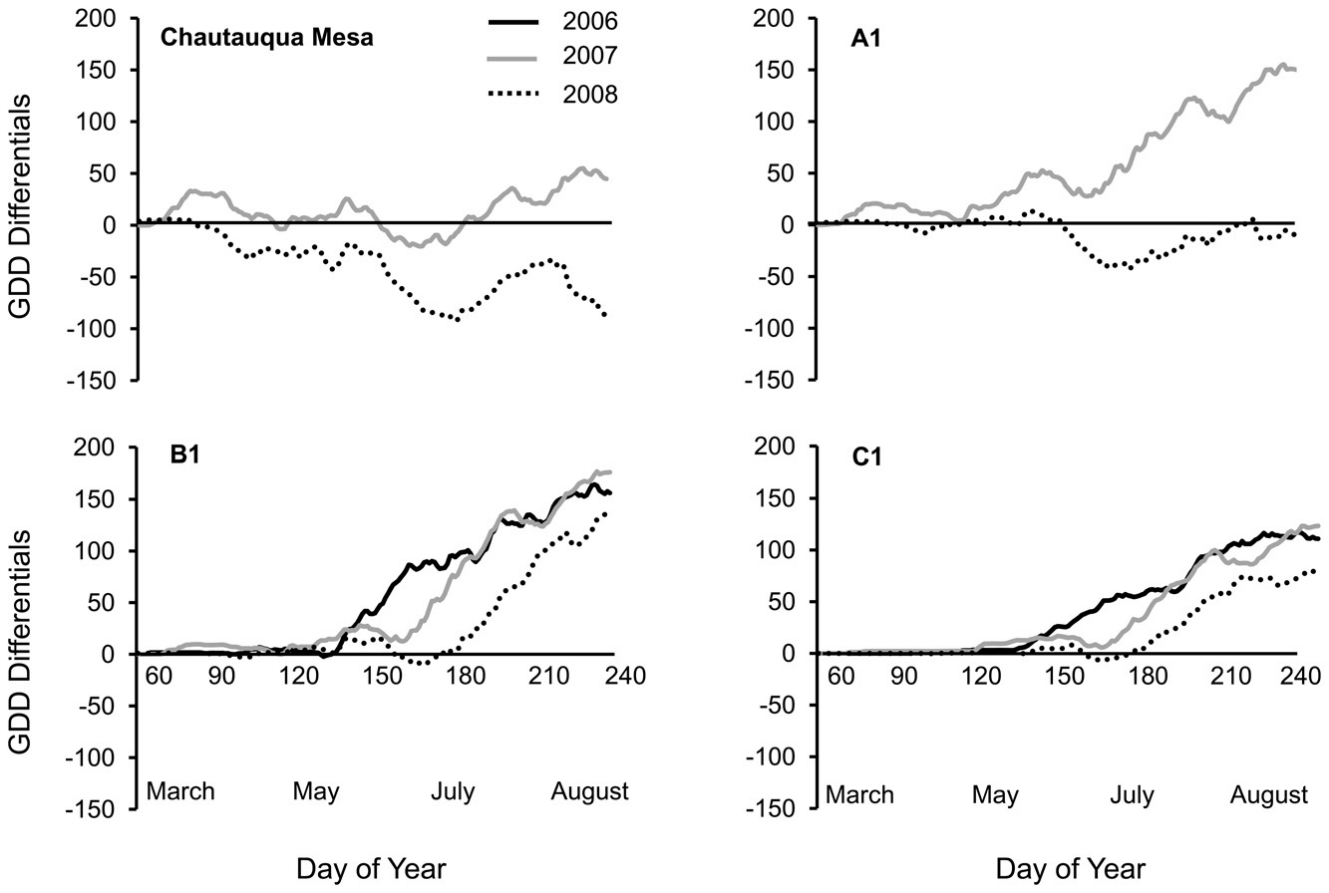
Recent seasonal growing degree day (GDD) differentials relative to 50 years prior. The running difference in GDDs is calculated as the accumulated GDDs for a given day on a particular year in the new survey (2006, 2007, 2008) minus the mean GDDs accumulated on the same date during the 1959–1960 surveys.

The total number of GDDs between surveys and the pattern with which they accumulated during a season (March 1st to August 31st) varied across sites and years (Figure 2). At Chautauqua Mesa, the 2007 season accumulated slightly more GDDs (+45) than the 1959–1960 average (1132 GDDs), while the 2008 season received fewer GDDs (−87). At A1, the 2007 season accumulated 150 GDDs more than the 1959–1960 average (747 GDDs), while 2008 accumulated slightly less than the average (−12). Sites B1 and C1 had similar overall GDD accumulation patterns, with all years having more GDDs than the 1959–1960 average. At B1, 2006, 2007 and 2008 experienced 157, 177, and 140 more GDDs, respectively, than the 500 GDD average for the 1959–1960 seasons. In turn, at C1, 2006, 2007 and 2008 experienced 111, 123, and 140 more GDDs, respectively, than the 200 GDD average for the 1959–1960 seasons.

While the number of GDDs was greater during the recent surveys at B1 and C1, the date at which the GDDs accumulation rates began to differ most from the 1959–1960 average came later each year for both sites. That is, at both sites, accumulated GDDs during the 1959–1960 and 2006 surveys began to differ at day 132, while in 2007 and 2008, the differences began to accumulate at days 165 and 175, respectively (Figure 2).

### Growing degree days and phenological advancement

Grasshoppers in higher-elevation communities required many fewer GDDs to reach adulthood than those at lower elevations. Averaging the number of GDDs required by species within a community, we found that increases in elevation led to communities requiring fewer GDDs for their associated species to become adults (r^2^ = 0.92, P = 0.02). The number of GDDs required by the communities declined by 0.25 GDDs per meter gain in elevation. Like the different communities, several species whose ranges span multiple sites (as illustrated by *A. clavatus*, *Camnula pellucida*, and *M. dodgei*) required fewer GDDs to reach adulthood at higher elevations compared to lower elevations (Table 2).

**Figure 3:**
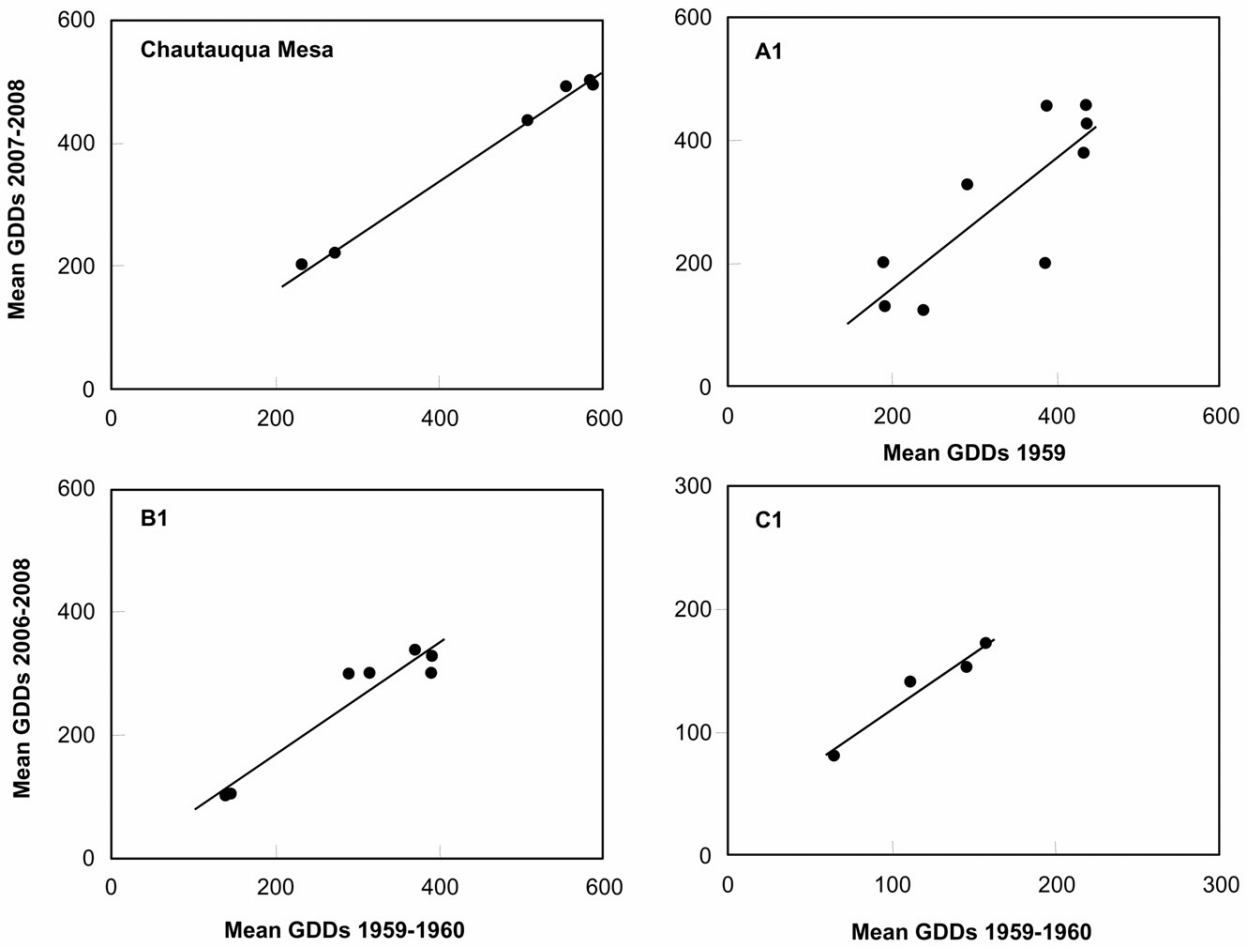
Thermal energy required by species to reach adulthood. Growing degree day (GDD) values associated with different grasshopper species at each of the four survey sites during Alexander's original survey (1959–1960) and the current resurvey (2006–2008). Note that original estimates of GDD values for A1 include data from1959 and that due to the much lower number of GDDs required by species at C1, the figure scales differ.

To address whether the GDDs required to reach adulthood at all sites were similar between Alexander and Hilliard's and our current survey, we regressed the average GDDs associated with the timing to adulthood of each species between both survey periods for each site. Consistent with the hypothesis that the number of GDDs are similar between survey periods, at each site we found that the slope of the relationship between the number of GDDs required in both studies did not differ from 1 (P>0.05). For example, at Chautauqua Mesa, where most species did not exhibit a consistent phenological advancement in their time to reach adulthood (Table 2), the GDDs required to reach adulthood in the early survey were strongly correlated with the number of GDDs required by grasshoppers in the recent survey (r^2^ = 0.99, P<0.0001; y = −17.11+0.89x; Figure 3). Although at A1, grasshopper communities varied greatly in their phenological responses (Table 2), the GDDs required for grasshoppers to reach adulthood during the original and current resurvey were also significantly correlated, although less so than at any other site (r^2^ = 0.66, P<0.008; y = −53.69+1.06x; Figure 3). Finally, grasshopper communities at B1 and C1, both of which showed significant phenological advancements (particularly in 2006 and in the species that become adults later in the season; Table 2) also demonstrated a significant relationship between the average number of GDDs required by grasshoppers to reach adulthood in the early and recent surveys (r^2^ = 0.92, P = 0.0007; y = −8.20+0.90x and r^2^ = 0.96, P = 0.02; y = 30.25+0.89x for B1 and C1, respectively; Figure 3).

## Discussion

Determining the causal mechanisms that lead species and communities to display differential phenological responses to climatic changes is of central interest in global change biology research; however, due to a lack of well documented and detailed phenology and climate data, this is difficult to address effectively. Because of these limitations, community-level studies have typically presented the occurrence of phenological events as they correlate to temperatures measured over a fixed period (months, seasons or years) [10], [13], [37], [38] and have often assumed that differences among species are a result of differences in life-history traits or other factors. With access to detailed climate data, a better approach to addressing the impact of warming on phenology involves relating phenological events to known temperature thresholds that must be met for that phenological event to occur [39], [40], [41]. The focus on GDDs can directly link timing and amount of heat input into the system with the corresponding phenological responses. In the following, we address the degree to which community-level phenological responses vary relative to the degree to which sites have warmed and the timing of warming. We also use GDDs to illustrate how the thermal energy required to reach adulthood have remained similar for the different grasshopper species within each community, and in turn, how changes in GDD accumulation rates explain the phenological advancement of species within communities.

### Degree of warming and phenological response across community and elevation

In this study, we found that the advancement in grasshopper phenology, measured as the first appearance of adults, was dependent upon the degree to which a site had warmed over the last 50 years. As in more extensive analyses of the climate data ([42]; McGuire *et al. in review*), this warming was found to be non-uniform across the elevational transect, with the lowest site experiencing little change and higher sites experiencing significant warming (Figure 1). In turn, the lowest site (Chautauqua Mesa) showed little evidence (if any) of an advancement in grasshopper phenology across the survey years and, although the next highest site (A1) showed some significant species level advancements, these advances were inconsistent across years and led to a lack of an overall significant phenological advancement being detected. The two highest sites differed from the lower sites as they showed either a consistent advancement across species during each year that varied in degree by year (B1) or an advancement across species during the first year which diminished considerably during the following years (C1). Although the phenological advancement of species at C1 diminished over time, like B1, a significant advancement in the timing to adulthood was detected. This study thus shows that warming and its associated community level responses can vary greatly along an elevational gradient, for which the extremes are only 50 km apart, but with an elevational difference of 1300 m. The importance and expected differences in warming and community responses along elevational gradients has been previously noted [43]. Finally, we note that these measured differences in phenological advancements, both across and within sites, were not explained by changes in the relative abundance of species over the 50 years prior.

### Factors influencing phenological advancement

Among the communities that showed the greatest phenological advancement (B1 and C1) we found that the degree to which species responded was not only site dependent, it was also time-of-season dependent; that is, at both B1 and C1, species that became adults later in the season displayed the greatest level of phenological advancement (Table 2). This finding is counter to many studies that have documented that earlier-appearing species tend to display the greatest advancements [12], [14]. As well, unlike a previous study on odonates that found that egg-diapausing dragonflies were less likely to respond phenologically to warming than nymphal diapausers [13], our study found that egg-diapausing grasshoppers (which were all of the grasshopper species included in this study) can readily respond to changes in climate. Our study thus highlights that phenological advancement may not only be a function of an organism's life-history characteristics [12], [13], phylogeny [44], or of a combination of environmental cues that influence the timing of their life-history events (timing of snow melt, temperature, day-length, etc.) [45], [46], [47]; but, as in other systems [6], it may also be attributed to the detailed seasonal timing of warming. At B1 and C1, for example, grasshoppers begin to enter adulthood around day of year 170 and 180, respectively, and by day of year 220 representatives of all grasshopper species at both sites have reached adulthood (Table 2). The GDD differentials show that warming at both sites, relative to the GDD accumulation patterns of the 1959 and 1960 surveys, is most apparent after days of year 132, 165 and 175, for years 2006, 2007 and 2008, respectively (Figure 2). Thus the “ramping-up” time of recent GDD accumulation patterns leads to warming impacting later-maturing species disproportionally by exposing them to more GDDs during their normal developmental windows than earlier species. In a recent field study where artificial heating units were used, researchers found evidence suggesting that later-maturing species of grasshoppers may be more likely to respond to warming than earlier-maturing species, and this may be due to differences in eco-physiological traits [48]. However, in the Guo *et al.* [48] study, it was not apparent whether the artificial warming treatments that were used exposed the grasshoppers to equal amounts of accumulated heat energy over time or whether, as in our study, later species showed greater advancements because they were exposed to more GDDs.

**Figure 4:**
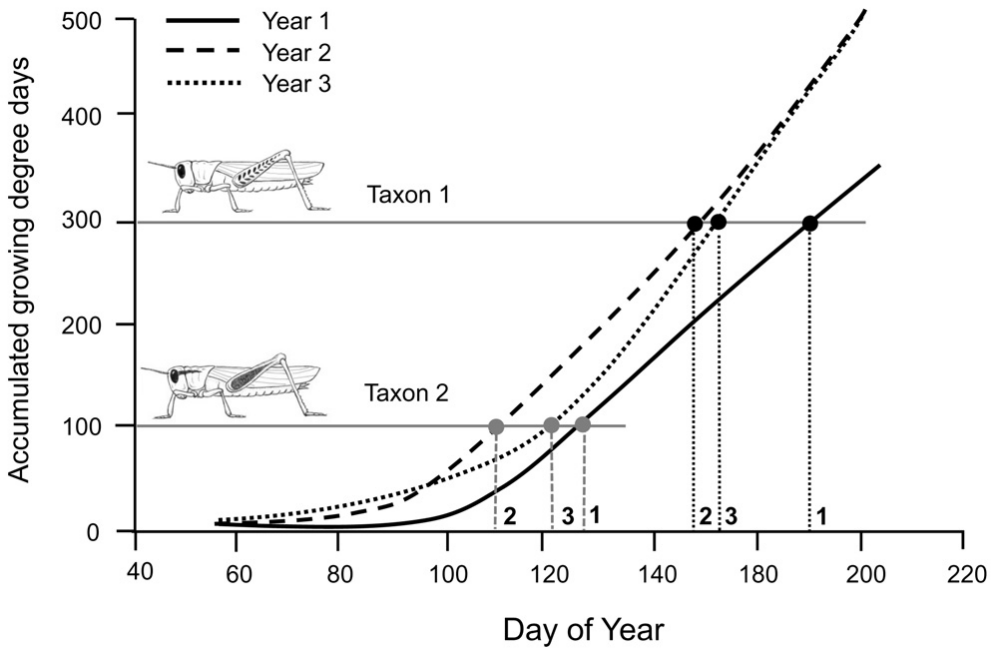
A descriptive model showing the effects of midseason warming. The degree to which early and late species show a phenological advancement may depend on the timing of warming and not necessarily the amount of warming. In year 2, relative to year 1, warming “ramps-up” early in the season, allowing both the early taxon (requiring 100 GDDs) and the late taxon (requiring 500 GDDs) to experience a significant phenological advancement. In year 3, however, the advancement occurs later in the season, leading to only the later species experiencing a significant advancement.

A significant year effect on the overall levels of phenological advancement was detected at B1 and C1. At B1, the average advancement across all species declined over the three years, from 19 days in 2006, to 16 days in 2007 and 13 days in 2008. At C1, all of the species at C1 showed a phenological advancement in their timing to adulthood during the first year (2006), while in the following year (2007) only the two species becoming adults latest in the season advanced, and in the final year (2008) only the species with adults appearing the latest showed any advancement (Table 2). The shift in the timing of warming during the 2006 to 2008 surveys at these two sites to later in the season (Figure 2) appears to have significantly affected changes in the community-wide levels of advancement. This study thus illustrates that similar levels of warming (as measured by accumulated number of GDDs) can have very different impacts on species within communities according to the seasonal timing of warming and that the seasonal timing of warming and its effects on phenology can be year dependent (Figure 4).

### Growing degree days and community responses

Within each community along this elevational gradient, the number of GDDs associated with when species became adults was similar across the previous (1959–1960) and current survey (Figure 3). This relationship was found for Chautauqua Mesa which showed minimal community-level phenological advancements, for A1 that showed some species advancements as well as delays and lack of responses, for B1 which displayed large and consistent advancements, and for C1 which showed clear advancements on the first year but which displayed declining advancements in the following years (Table 2). That the number of required GDDs were similar between surveys within sites that differed in levels of phenological advancement suggests that the lack of an overall advancement in grasshopper phenology was largely due to the required GDDs being reached at similar times as in the previous survey (Chautauqua Mesa) and that advancements at other sites were due to the required GDDs being reached earlier than in the previously survey (B1 and C1; Figure 2).

We have shown that changes in the timing to adulthood of grasshoppers can be explained by differential changes in warming experienced by the different sites, the seasonal timing of warming and year-to-year changes in this seasonal timing. As well, we have shown that the required temperature thresholds (GDDs) for grasshoppers to become adults have remained unchanged over the last fifty years. Taken together, these findings allow us to conclude that grasshoppers in the Front Range of Colorado are being affected by recent warming patterns and that their responses likely reflect developmental plasticity rather than adaptation. However, a consistent change in the thermal environment to which species are exposed may have an effect on other characteristics such as body size, the number of generations present per year and fecundity [49], [50], and ultimately to changes in demography and distributions [9], [26]. Over time, these changes may hasten evolved responses to warming conditions [51], [52].

### Community differences in required growing degree days

That grasshoppers at higher elevations (which have shorter growing seasons and lower average daily temperatures) require fewer GDDs to complete development (Table 2) has been previously documented [53]. Grasshoppers at higher elevations may develop using fewer GDDs by: 1) changing their thermoregulatory behaviors (allowing them to reach proportionally higher than ambient temperatures) [21], [54]; 2) having faster developmental rates (that may be a result of increases in consumption or assimilation rates) [55]; and/or 3) by adopting a smaller adult body size at adulthood [56], [57]. While these behavioral and physiological changes may be partially environmentally induced, there is also evidence that these changes may have a strong genetic component [54], [58].

The multispecies approach used in this study showed that species found at two or more sites consistently require fewer GDDs at higher elevations (Table 2). In addition, on a community-wide level, we found that from the prairie to the subalpine, grasshopper communities require 0.25 fewer GDDs per meter increase in elevation. If the required number of GDDs to complete development during a season limits the upper range of species, then increased warming should allow species to expand their distribution higher up the mountain. For example, at B1, relative to the 1959–1960 survey, the number of available GDDs per season has increased by 28–35% while at C1 the number of available GDDs has increased by 55–70%. While no new species have been detected at these sites, future surveys will focus on range expansions. Although the focus of this study was on grasshopper communities, such large changes in accumulated GDDs will likely have impacts on other groups of organisms as well.

Future work should also determine the degree to which the lower number of required GDDs associated with grasshoppers at higher elevations may impact the way that communities respond to future climate change. For example, an increase of 30 GDDs in a season could speed up development of *M. dodgei* by 13% at A1 where 240 GDDs are required, by 22% at B1 where 137 GDDs are required and by nearly 50% at C1 where only 61 GDDs are required. We plan future work to model how the amount of warming, timing of warming and required GDDs may lead communities at different elevations to respond differentially to projected warming (see [59], [60]).

### Conclusions

In this study, we assembled a multispecies and multisite dataset that documents warming over a 50 year time frame, along with seasonal GDD accumulation rates and grasshopper phenology data. Our analyses provide clear evidence that variation in the amount and timing of warming over the grasshopper growing season explains the vast majority of phenological variation, a result that does not require us to invoke differences that are due to life-history traits. For example, while grasshopper species associated with Chautauqua Mesa, a site that has not warmed significantly over the last 50 years, showed little advancement; grasshoppers associated with the sites that have experienced the most warming (B1 and C1) displayed the greatest levels of phenological advancement. Still, all four communities in this study displayed dramatically different responses to recent warming (Table 2). In turn, within the communities that displayed the greatest advancement (B1 and C1), progressively later seasonal warming led to later-developing species showing stronger phenological advancements than earlier-developing species. Although we did not find evidence for adaptation to climatic changes over time, we did find that communities at higher elevations require significantly fewer GDDs to develop to adulthood than those at lower elevation.

Taken together, we believe that our results move past simple correlation and provide a stronger process-oriented and predictive framework for understanding community-level phenological responses to climate change. This framework is also extendable well beyond grasshoppers. For example, we suggest that before invoking other possible explanations for differential phenological response among species in a community, it is important to first accurately determine when warming is occurring in relation to seasonal developmental timing. This will be important for many groups of organisms and may provide insight into why certain taxa respond to warming whereas others do not. In addition, as the utility of linking temperature, GDDs, and phenological development has shown in this study (see also [39], [41], [59], [60], [61]), we suggest that through incorporation of GDDs into analyses and models of species and community responses, it will be possible to predict more precisely future phenological advancements based on different warming scenarios.
